# Des taches brunes à méditer

**DOI:** 10.11604/pamj.2015.22.89.7845

**Published:** 2015-10-01

**Authors:** Youssef Kort, Naziha Khammassi

**Affiliations:** 1 Faculté de Médecine de Tunis, Service de Médecine Interne, Hôpital Razi, la Manouba 2010, Tunisie

**Keywords:** syndromes paranéoplasiques cutanés, kératose séborrhéique, Leser-Trélat, paraneoplastic skin syndrome, seborrheic keratosis, Leser-Trelat

## Image en medicine

Un syndrome paranéoplasique est une manifestation biologique ou clinique d'un cancer liée au cancer mais sans en être la conséquence directe ou de celle de ses métastases. Les syndromes paranéoplasiques cutanés en sont l'exemple le plus visible. Patient âgé de 72 ans suivi pour une hypertension artérielle et qui rapportait l'apparition puis l'extension rapide (en quelques mois) de lésions cutanées hyper pigmentées du tronc et de l'abdomen. Aucune autre symptomatologie n’était rapportée. L'examen cutané notait au niveau de la face, du tronc, de l'abdomen, du dos et des cuisses des dizaines de lésions verruqueuses et brunâtres, de forme grossièrement ovalaires mesurant de 0.5 à2 cm de diamètre réalisant un aspect typique de kératose séborrhéique. Le diagnostic différentiel d'une tumeur brune peut se poser avec un lentigo solaire, un mélanome ou des verrues. Un bilan extensif à la recherche d'un cancer a été réalisé (examen ORL avec naso fibroscopie, fibroscopie digestive haute et basse et une TDM thoraco-abdomino-pelvienne) et qui s'est révélé négatif. A deux ans de recul, aucun cancer n'a été décelé. L'apparition et la multiplication rapide de kératoses séborrhéiques définie le signe de Leser-Trélat (SLT) qui peut être potentiellement paranéoplasique (surtout adénocarcinome gastrique). Les syndromes paranéoplasiques dermatologiques (SPD) sont classés en 3 catégories: Les SPD obligatoires tel que le signe de Bazex constamment rattaché à un cancer, les SPD facultatifs qui peuvent être liés ou pas à un cancer et les SPD controversés tels que le SLT ou le lien avec un cancer n'a pas été formellement établi.

**Figure 1:**
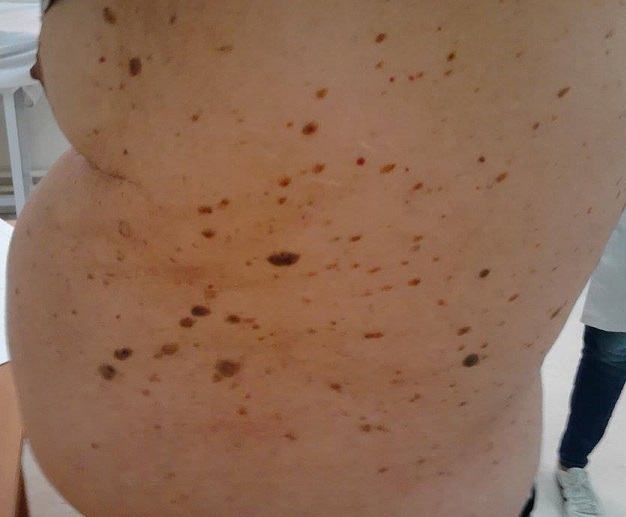
Plusieurs lésions verruqueuses et brunâtres, de forme grossièrement ovalaires au niveau de l'abdomen et du dos

